# The rs5888 single nucleotide polymorphism in scavenger receptor class B type 1 (SCARB1) gene and the risk of premature coronary artery disease: a case-control study

**DOI:** 10.1186/s12944-016-0176-9

**Published:** 2016-01-12

**Authors:** Hamidreza Goodarzynejad, Mohammadali Boroumand, Mehrdad Behmanesh, Shayan Ziaee, Arash Jalali

**Affiliations:** Department of Cardiac Research, Tehran Heart Center, Tehran University of Medical Sciences, Tehran, Iran; Department of Molecular Pathology, Tehran Heart Center, Tehran University of Medical Sciences, North Karegar Ave.and Jalal-Al-Ahmad cross, Tehran, Iran; Department of Genetics, School of Biological Sciences, Tarbiat Modares University, P.O. Box: 14115-154, Tehran, Iran; Department of Laboratory Medicine and Pathology, Tehran Heart Center, Tehran University of Medical Sciences, Tehran, Iran

**Keywords:** Coronary artery disease, Premature atherosclerosis, Coronary angiography, Scavenger receptor class B type I, rs5888 polymorphism

## Abstract

**Background:**

Several single nucleotide polymorphisms (SNPs) in lipid transport genes have been shown to be associated with premature coronary artery disease (PCAD). The scavenger receptor BI (SCARB1) is a key component of the reverse cholesterol transport and lipid metabolism. We aimed to examine the relationship between the rs5888 SNP within SCARB1and the risk of angiographically determined PCAD.

**Methods:**

We used an age cut-off of 55 years for women and 45 years for men to define PCAD. Five-hundred and five patients with newly diagnosed angiographically documented PCAD (≥50 % luminal stenosis of any coronary vessel) as case group compared with 546 controls (subjects with no luminal stenosis at coronary arteries). The severity of CAD was determined by vessel score as well as Gensini score. A real-time polymerase chain reaction (PCR) and High Resolution Melting (HRM) analysis was used to distinguish between genotypes.

**Results:**

T allele as compared to C allele was associated with increased odds of PCAD in total population (adjusted OR = 1.3, 95 % CI = 1.0 to 1.5; *p* = 0.020), and in women (adjusted OR = 1.3, 95 % CI = 1.0 to 1.8; *p* = 0.037), but not in men (adjusted OR = 1.2, 95 % CI = 0.9 to 1.5; *p* = 0.311). There was also no significant association between the examined polymorphism and the severity of CAD in whole or in men or women subgroups.

**Conclusions:**

Our findings suggest that the SNP (rs5888) within SCARB1 is independently associated with PCAD in a sex-dependent manner.

## Background

The occurrence of coronary artery disease (CAD) in younger subjects, although infrequent, causes devastating effect on the more active lifestyle of this group of patients and may result in premature death [1]. Patients with premature CAD (PCAD), defined as the presence of coronary artery atherosclerotic lesion in men ≤45 year and women ≤55 years, have different risk factor profiles, clinical presentations, and prognoses than older patients [2]. Even though the identification of risk factors for PCAD is of essential importance for the understanding of its origins and for establishing preventive strategies, the mechanisms and predictors of PCAD are still not completely understood [3]. Risk factors for CAD are both environmental and genetic. In patients with PCAD, the role of genetic risk factors is expected to be more important than that of environmental factors [4]. Over the past recent years, several single nucleotide polymorphisms (SNPs) in lipid transport genes have been shown to be associated with PCAD [5–9].

Scavenger receptor class B type1 (SCARB1, also called SR-BI) is a multiligand cell surface receptor expresses both in macrophages and in the liver, indicating a major role for in the clearance of excess cholesterol from the body [10]. This membrane protein facilitates the uptake of cholesteryl esters from high-density lipoprotein cholesterol (HDL-C) and drives cholesterol from tissues to the liver in the various stages of reverse cholesterol transport pathway. This makes SCARB1 gene as an attractive candidate gene for CAD. The SCARB1 gene is located on 12q24.31 encompassing 13 exons and spans approximately 75 kb. Various SCARB1 polymorphisms in humans have been shown to be associated with altered serum lipid profile but their influence on CAD development or severity is still unclear [11]. One exonic SNP (rs5888) within SCARB1, has been linked to lower SR-BI protein expression and function [12]. This SNP is a “C” to “T” substitution at cDNA position 1050 base position on exon 8, and a coding-synonymous polymorphism (A350A) which was initially found by Acton et al. [13]. Several studies on rs5888 polymorphism and the risk of CAD have been previously published with contradictory results [14–18]. There is even controversy about which allele confers increased risk. Wu et al. [14] found that the individuals with TT genotype were associated with increased CAD risk, whereas others reported that the T allele or the T allele carrier was associated with decreased risk of CAD [15–18]. Moreover, they have investigated this association amongst general CAD populations and there is no study, that we are aware of, studying this association in PCAD patients. Thus, the aim of the present study was to examine the relation of rs5888 SNP with the presence and severity of angiographically determined PCAD in an Iranian coronary population.

## Methods

### Study participants and coronary angiograms

A prior study has used an age cut-off of 55 years for women and 45 years for men to define “young” patients with CAD [2]. To define PCAD, the same age definition was used in this study. This age- and sex-matched case–control study was conducted on 560 patients with newly diagnosed angiographically documented PCAD and an equal number of controls with normal coronary arteries. Cases were selected consecutively from those who, between August 2009 and May 2011, underwent coronary angiography at the catheterization laboratory of our center because of symptoms related to CAD or the results of non-invasive tests. Unrelated matched controls were selected from a pool of 1547 young patients (419 men and 1128 women) who, between June 2004 and July 2011, consecutively admitted for elective coronary angiography at our hospital. Cases and controls were matched for age within two years. Exclusion criteria included previous history of acute myocardial infarction (MI), stent implantation, cardiopulmonary resuscitation (CPR), and coronary artery bypass graft surgery. Also, patients on lipid-lowering drugs and those with minimal CAD (coronary lesions with less than 50 % luminal stenosis) were excluded. The study protocol was approved by the local ethical committee. Written informed consent was obtained from all patients in agreement with the Declaration of Helsinki for research involving human subjects explicitly provided permission for DNA analyses and gathering the relevant clinical data.

Coronary angiographies were performed by the percutaneous femoral approach using standard techniques. The presence and severity of CAD was determined by clinical vessel score. All cases showed the evidence of atherosclerosis i.e., ≥ 50 % luminal stenosis in at least one coronary artery or major branch segment in their epicardial coronary tree. Controls had no luminal stenosis at coronary angiograph. The severity of CAD was also determined by a semi-quantitative scoring system (Gensini score) which has been previously described [19]. Two cardiologists, blind to the study results, interpreted all angiograms. Definitions for analyzed risk factors of CAD including, body mass index (BMI), dyslipidemia, hypertension, cigarette smoking, opium addiction, and diabetes have been reported elsewhere [20, 21].

### Sample collection and DNA extraction

From June 2004 to July 2011, for the purpose of creating a “DNA-bank of patients with PCAD and controls”, all young patients (age ≤45 years for men, and age ≤55 years for women at the disease onset) admitted for elective coronary angiography at our center were asked to provide a sample of whole blood for DNA extraction Peripheral venous blood samples were collected from an antecubital vein after 12 h overnight fasting of participants. Upon arrival at the laboratory, each participant provided about 15-mL venous blood sample. 5 mL blood was collected in plain tubes and used for biochemical assays immediately; the rest of the sample (10 ml) placed in ethylenediamine tetraacetic acid (EDTA)-containing tubes and stored deep-frozen until later use. Genomic DNA was extracted from leukocytes using the buffy coat of the stored EDTA whole blood samples. DNA extraction carried out using the standard ‘salting out’ method. DNA quantity was evaluated by calculating absorbance at λ = 260 nm, and the quality was assessed by a ratio of λ = 260/280 nm being close to 1.8. The purified DNA was stored in Tris-EDTA buffer (pH 8.0) at –70 °C until further analysis.

### PCR amplification and genotype analysis

**Fig. 1:**
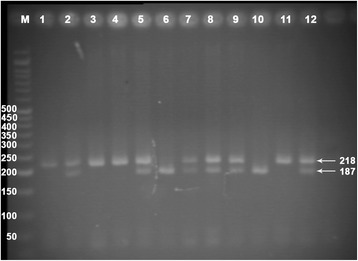
Agarose gel electrophoresis for the rs5888 polymorphism of scavenger receptor class B type 1 (SCARB1) gene. The 218-bp PCR product of SCARB1 rs5888 (C > T) was digested by Hin1I (BsaHI) restriction enzyme. The C allele codes for the presence of the Hin1I site and the T allele codes its absence. The wild-type CC variant produced two fragments with 187 bp and 31 bp while heterozygote CT produced 3 fragments of 218 bp, 187 bp, and 31 bp. The mutated homozygous variant TT produced one fragment of 218 bp. The 31 bp fragment was not visible in the gel due to its fast migration speed. M, 50 bp marker ladder; Lanes 6 and 10, CC genotype; Lanes 2, 5, 7, 8, 9, and 12, CT genotype; Lanes 1, 3, 4, and 11, TT genotype

Real-time polymerase chain reaction (PCR) and High-Resolution Melting (HRM) analysis was applied for the genotype analysis using Corbett Rotor-Gene 6000 real-time rotary analyzer (Corbett Life Science Pty. Ltd., Mortlake, NSW, Australia). One set of primers based on common HRM specifications was designed using Beacon Designer software version 7.0 (Premier Biosoft International, Palo Alto, CA, USA) and synthesized by Bioneer Inc. (Daejeon, Korea). The forward primer was 5′- CTTGTTTCTCTCCCATCCTCA -3′ and the reverse was 5′- GAGTGTGCCTCCTGGTTAG -3′. The final reaction mixture contained Type-it HRM Master Mix (QIAGEN NV, Venlo, Netherlands) with intercalating DNA-binding Evagreen dye (Biotium, Inc. Hayward, CA, USA), DNA polymerase (5U/ml), primer mix (0.4 μM of each primer), genomic DNA (50 ng), and RNase Free Water (QIAGEN NV) in a total volume of 20 μl. The PCR cycling conditions consisted of an initial denaturation at 95 °C for 15 min, followed by 37 cycles of denaturation (96 °C for 20 s), primer annealing (62 °C for 20 s), and extension (72 °C for 15 s). One positive control for each genotype (CC, CT, and TT) and one appropriate negative control were included in each run. The positive controls were verified by using the PCR with restriction fragment length polymorphism (RFLP) technique as described previously with minor modification [22]. The genotypes were separated by electrophoresis on 2 % agarose gel stained with ethidium bromide (Fig. 1) and also confirmed by direct DNA sequence analysis.

Following the PCR amplification steps, melt curves for the products were generated by heating in 0.1 °C increments at a rate of 2 s per each step over the temperature range from 65 to 95 °C. The HRM data were analyzed using the Rotor-Gene Q software package supplied with the instrument. Sequence variations were distinguished from wild-type samples by the different shapes of normalized and temperature-shifted melting curves. In samples which failed to give an interpretable HRM pattern (10 %), the PCR-RFLP method was used for genotyping and reconfirmed by direct sequencing in 15 samples.

### Statistical analysis

Continuous variables are reported as “mean ± standard deviation” and categorical variables as “numbers and percentage”. Variables were tested for normality by visually inspection of box-plots and by using Kolmogorov–Smirnov normality test. Gensini score, which was skewed, presented as median and inter-quartile range (25^th^ to 75th percentiles). The case and control groups were compared using the independent two-sample Student *t*-test (or Mann-Whitney *U*-test if required) for the continuous variables and the chi-square test (or the Fisher exact test, as appropriate) for the categorical variables. Due to the highly skewed distribution of Gensini scores, to compare Gensini score among rs5888 genotypes, the Kruskal-Wallis analysis was chosen and subjects with normal coronary arteries (Gensini score = 0, *n* = 546) were excluded to ensure any observed effect on graded severity was not being driven by those without any CAD in whom risk allele frequency is expected to be significantly lower.

Logistic regression model was constructed to test the independent relationship between the rs5888 variants and the presence of CAD with the SNP coded as 0, 1, or 2 based on the number of risk (T) alleles. Analyses were repeated after adjusting for diabetes mellitus, cigarette smoking, opium addiction, hypertension, and family history of CAD as well as serum HDL-C, triglyceride, and LDL-C in an established multivariable model. Odds ratio (OR) and 95 % confidence intervals (CI) were calculated in all patients and in males and females separately. Moreover, a linear multiple regression analysis with the presence of CAD severity (Gensini score as a continuous variable) as the dependent variable, was performed. The following were tested as potential risk factors: diabetes mellitus, cigarette smoking, opium addiction, hypertension, and family history of CAD as well as BMI and serum levels of HDL-C, triglyceride, and LDL-C. Non-normal variables, such as Gensini scores, were natural log-transformed before entering the model. Additionally, a multiple ordinal logistic regression model, with number of diseased vessels as the dependent variable, was established with adjusting for aforementioned potential risk factors. A generalized linear model (GLM) was used to analyze the association between the genotypes and lipid profile adjusted for age, sex, BMI, and cigarette smoking. A *p* value ≤ 0.05 was considered statistically significant. All Statistical analyses were done by PASW Statistics for Windows, Version 18.0. (Chicago: SPSS Inc.).

## Results

Sixty-nine patients (55 cases and 14 controls) were excluded due to missing data or genotyping failure, resulting in 1051 patients entered in the statistical analyses including 505 cases, and 546 controls. The mean age of the study participants was 46 ± 6 years and 45.4 % were men. Baseline characteristics of the study sample stratified by gender and CAD status are presented in Table 1 and 2, respectively. The overall prevalence of cardiovascular risk factors including, hypertension, diabetes mellitus, cigarette smoking, dyslipidemia, and family history of CAD were high. The women had a greater prevalence of hypertension, diabetes mellitus, and dyslipidemia as compared to men but cigarette smoking and opium addiction were much more frequent in men than in women. There was no significant difference in the prevalence of family history of CAD between men and women (Table 1). As seen in Table 2, the prevalence of diabetes, family history of CAD, hyperlipidemia, and cigarette smoking as well as hypertension and opium addiction were significantly higher in CAD group whereas there was no statistically significant difference in age, sex, and BMI between two groups.

**Table 1:** Baseline characteristics of the study population stratified by sex

	All (*n* = 1051)	Men (*n* = 477)	Women (*n* = 574)	*P*-value^*^
Age (years)	45.6 ± 6.0	41.0 ± 3.5	49.4 ± 4.9	<0.001
Body mass index (kg/m^2^)	29.7 ± 5.7	28.1 ± 4.4	31.1 ± 5.3	<0.001
Cigarette smoking				<0.001
Current smoker	213 (20.3)	188 (39.4)	25 (4.4)	
Ex-smoker	97 (9.2)	82 (17.2)	15 (2.6)	
Non-smoker	741 (70.5)	207 (43.4)	534 (93.0)	
Opium addiction	99 (9.4)	91 (19.1)	8 (1.4)	<0.001
Hypertension	456 (44.3)	126 (26.4)	339 (59.1)	<0.001
Diabetes	240 (22.8)	53 (11.1)	187 (32.6)	<0.001
Dyslipidemia	673 (64.0)	290 (60.8)	383 (66.7)	0.046
Family history of CAD	357 (34.0)	157 (32.9)	200 (34.8)	0.511
Fasting blood glucose (mg/dl)	116.7 ± 50.1	106.8 ± 41.1	124.8 ± 52.2	<0.001
Triglyceride (mg/dl)	182.8 ± 116.4	193.3 ± 123.1	174.1 ± 109.9	0.008
HDL-cholesterol (mg/dl)	42.2 ± 11.7	38.5 ± 10.5	45.2 ± 11.8	<0.001
LDL-cholesterol (mg/dl)	117.0 ± 40.3	113.4 ± 37.1	119.9 ± 42.5	0.011
Total cholesterol (mg/dl)	186.8 ± 48.6	182.2 ± 47.7	190.6 ± 49.5	0.005
Creatinine (mg/dl)	0.9 ± 0.6	1.0 ± 0.8	0.8 ± 0.4	<0.001
LVEF (%)	53.8 ± 9.0	52.7 ± 9.7	54.7 ± 8.3	<0.001

Data are presented as mean ± SD or n (%)

*CAD* Coronary artery disease, *LVEF* left ventricular ejection fraction

^*^ *P*-values for men vs. women

**Table 2:** Baseline characteristics in the study population based on coronary artery disease presence

Variables	non-CAD (*n* = 546)	CAD (*n* = 505)	*P* value
Age (years)	45.6 ± 6.1	45.6 ± 5.9	0.922
Body mass index (Kg/m^2^)	29.7 ± 4.9	29.7 ± 5.4	0.996
Male sex	243 (44.5)	234 (46.3)	0.551
Diabetes mellitus	74 (13.6)	166 (32.9)	<0.001
Hypertension	192 (35.2)	273 (54.1)	<0.001
Smoking status			0.001
Current smoker	87 (15.9)	126 (25.0)	
Ex-smoker	48 (8.8)	49 (9.7)	
Non-smoker	411 (75.3)	330 (65.3)	
Family history of CAD	148 (27.1)	209 (41.4)	<0.001
Dyslipidemia	315 (57.7)	342 (72.6)	<0.001
Triglyceride (mg/dl)	173.4 ± 114.3	193.0 ± 118.0	0.007
HDL-cholesterol (mg/dl)	43.9 ± 11.5	40.2 ± 11.7	<0.001
LDL-cholesterol (mg/dl)	116.4 ± 36.3	117.6 ± 44.2	0.945
Total cholesterol (mg/dl)	186.9 ± 42.8	186.8 ± 54.3	0.996
Fasting blood glucose (mg/dl)	106.1 ± 36.7	128.3 ± 60.2	<0.001
Creatinine (mg/dl)	0.9 ± 0.6	0.9 ± 0.6	0.981
LVEF (%)	55.7 ± 8.0	51.7 ± 9.6	<0.001

Data are presented as mean ± SD or *n* (%)

*CAD* Coronary artery disease, *LVEF* left ventricular ejection fraction

Allele and genotype frequencies for the SCARB1 (rs5888) polymorphism in the case and control groups, separated by sex, are shown in Table 3. There was no statistically significant difference in the distribution of rs5888 genotypes between two groups and in men and women subgroups after stratification by sex. Table 4 shows that no associations were detected between SCARB1 rs5888 (C > T) genotypes and lipid profile including total cholesterol, HDL-C, LDL-C, and triglyceride levels. Even after analyzing data separately for men or women, the rs5888 SNP in SCARB1 gene was not associated with lipid profile in the subgroup of men or women (data not shown).

**Table 3:** Genotype and allele frequencies of rs5888 polymorphism in the SCARB1 gene according to CAD status in whole study group and in subgroups separated by gender

	non- CAD patients			CAD patients			*P*-value^*^		
	All (*n* = 546)	Male (*n* = 243)	Female (*n* = 303)	All (*n* = 505)	Male (*n* = 234)	Female (*n* = 271)	All	Male	Female
rs5888 (C > T)							0.163	0.781	0.143
CC	195 (35.7)	88 (36.2)	107 (35.3)	162 (32.1)	81 (34.6)	81 (29.9)			
CT	281 (51.5)	121 (49.8)	160 (52.8)	259 (51.3)	115 (49.1)	144 (53.1)			
TT	70 (12.8)	34 (14.0)	36 (11.9)	84 (16.6)	38 (16.2)	46 (17.0)			
CT + TT	351 (64.3)	155 (63.8)	196 (64.7)	343 (67.9)	153 (65.4)	190 (70.9)	0.214	0.715	0.167
Alleles							0.082	0.544	0.070
C	671 (61.4)	297 (61.1)	374 (61.7)	583 (57.7)	277 (59.2)	306 (56.5)			
T	421 (38.6)	189 (38.9)	232 (38.3)	427 (42.3)	191 (40.8)	236 (43.5)			

^*^ *p*-values for non-CAD vs. CAD

Data are presented as n (%)

CAD, Coronary artery disease; SCARB1, scavenger receptor class B type 1

**Table 4:** Lipid profile in total population, and in case and control groups according to rs5888 (C > T) genotypes in SCARB1 gene

	CC	CT	TT	*P* value*
All participants	(*n* = 357)	(*n* = 540)	(*n* = 154)	–
Triglyceride (mg/dl)	183.6 ± 108.7	179.6 ± 113.7	192.5 ± 140.5	0.572
HDL cholesterol (mg/dl)	41.8 ± 11.6	42.6 ± 12.1	41.4 ± 10.4	0.569
LDL cholesterol (mg/dl)	119.5 ± 40.2	115.9 ± 40.3	114.9 ± 40.4	0.351
Total cholesterol (mg/dl)	188.5 ± 51.1	186.2 ± 47.3	185.1 ± 47.2	0.691
Non-CAD group	(*n* = 195)	(*n* = 281)	(*n* = 70)	–
Triglyceride (mg/dl)	175.7 ± 113.2	166.0 ± 99.0	196.4 ± 162.6	0.065
HDL cholesterol (mg/dl)	43.5 ± 12.6	44.3 ± 11.0	43.8 ± 9.5	0.853
LDL cholesterol (mg/dl)	120.6 ± 36.2	113.0 ± 34.6	118.2 ± 41.6	0.054
Total cholesterol (mg/dl)	189.4 ± 44.0	183.8 ± 40.7	191.5 ± 46.6	0.108
CAD group	(*n* = 162)	(*n* = 259)	(*n* = 84)	–
Triglyceride (mg/dl)	193.3 ± 102.5	194.2 ± 126.4	189.1 ± 119.4	0.887
HDL cholesterol (mg/dl)	39.7 ± 10.0	40.8 ± 12. 9	39.3 ± 10.7	0.499
LDL cholesterol (mg/dl)	118.2 ± 44.5	119.0 ± 45.4	112.0 ± 39.4	0.470
Total cholesterol (mg/dl)	189.4 ± 58.9	188.8 ± 53.4	179.6 ± 47.5	0.350

*Adjusted for age, sex, cigarette smoking, and body mass index (BMI)

Data are presented as mean ± SD

CAD, Coronary artery disease; SCARB1, scavenger receptor class B type 1

After applying a multiple logistic regression model with adjusting for diabetes, hypertension, cigarette smoking, opium addiction, BMI, and family history of CAD as well as triglyceride, HDL-C, and LDL-C, rs5888 polymorphism was found to be associated with the presence of PCAD in total population and in women but not in men (Table 5). T allele as compared to C allele was associated with increased odds of PCAD in total population (adjusted OR = 1.3, 95 % CI = 1.0 to 1.5; *p* = 0.020), and in women (adjusted OR = 1.3, 95 % CI = 1.0 to 1.8; *p* = 0.037), but not in men (adjusted OR = 1.2, 95 % CI = 0.9 to 1.5; *p* = 0.311). Among total population, those with TT genotype had 1.7-fold increased adjusted odds of PCAD compared to those with CC genotype (*p* = 0.013). When data were analyzed separately for men or women, SCARB1 (rs5888) TT genotype as compared to CC genotype was associated with increased odds of PCAD in women (adjusted OR = 2.1, 95 % CI = 1.2 to 4.0; *p* = 0.014), but not in men (adjusted OR = 1.3, 95 % CI = 0.7 to 2.5; *p* = 0.336).

**Table 5:** Unadjusted and adjusted odds ratios for the effect of rs5888 genotypes on premature coronary artery disease in whole and in men and women separately

	Unadjusted		Adjusted*	
rs5888 (C > T)	Odds ratio (95 % CI)	*p*-value	Odds ratio (95 % CI)	*p*-value
All participants		0.164		0.046
CC	1.0 (Ref.)	-	1.0 (Ref.)	–
CT	1.1 (0.9 to 1.5)	0.448	1.2 (0.9 to 1.6)	0.204
TT	1.4 (1.0 to 2.1)	0.058	1.7 (1.1 to 2.6)	0.013
CT + TT	1.2 (0.9 to 1.5)	0.214	1.3 (1.0 to 1.7)	0.077
Allele T vs. C	1.2 (1.0 to 1.4)	0.082	1.3 (1.0 to 1.5)	0.020
Male sex		0.781		0.583
CC	1.0 (Ref.)	–	1.0 (Ref.)	–
CT	1.0 (0.7 to 1.5)	0.874	1.2 (0.8 to 1.8)	0.448
TT	1.2 (0.7 to 2.1)	0.491	1.3 (0.7 to 2.5)	0.336
CT + TT	1.1 (0.7 to 1.6)	0.715	1.2 (0.8 to 1.9)	0.348
Allele T vs. C	1.1 (0.8 to 1.4)	0.544	1.2 (0.9 to 1.5)	0.311
Female sex		0.146		0.044
CC	1.0 (Ref.)	–	1.0 (Ref.)	–
CT	1.2 (0.8 to 1.7)	0.354	1.1 (0.7 to 1.8)	0.551
TT	1.7 (1.0 to 2.9)	0.050	2.1 (1.2 to 4.0)	0.014
CT + TT	1.3 (0.9 to 1.8)	0.167	1.3 (0.9 to 2.0)	0.202
Allele T vs. C	1.2 (1.0 to 1.6)	0.070	1.3 (1.0 to 1.8)	0.037

*Adjusted for diabetes, hypertension, cigarette smoking, opium addiction, BMI, and family history of CAD as well as triglyceride, HDL-C, and LDL-C

CAD, coronary artery disease; BMI, body mass index; CI, confidence interval; HDL-C, high density lipoprotein cholesterol; LDL-C, low density lipoprotein cholesterol; Ref., the reference category

Table 6 shows the association between the studied polymorphism and the severity of CAD with respect to vessel score and Gensini score. The distribution of vessel score was statistically similar among different genotypes (adjusted *p* = 0.309). Additionally, in total population, the median and inter-quartile range for Gensini score was not significantly different among the CC (42, 20 to 65), CT (42, 23 to 75), and TT (37, 20 to 64) genotypes (adjusted *P* = 0.540). There was also no significant association between the examined polymorphism and the severity of CAD within men or women subgroups.

**Table 6:** The genotypes of rs5888 single nucleotide polymorphism in association with CAD severity

	CC (*n* = 357)	CT (*n* = 540)	TT (*n* = 154)	*P*-value	*P*-value^*^
Vessel score, n (%)				0.178	0.247
0	195 (54.6)	281 (52.0)	70 (45.3)		
1VD	72 (20.2)	117 (21.7)	46 (29.9)		
2VD	40 (11.2)	74 (13.7)	22 (14.3)		
3VD	50 (14.0)	68 (12.6)	16 (10.4)		
Gensini score, median (25th to 75th percentiles), n^a^.					
All	42 (20 to 65), 162	42 (23 to 75), 259	37 (20 to 64), 84	0.449	0.572
Males	43 (26 to 77), 81	40 (23 to 67), 115	36 (25 to 61), 38	0.816	0.697
Females	40 (20 to 64), 81	45 (23 to 78), 144	38 (18 to 66), 46	0.233	0.398

*0* no coronary lesion, *1VD* one-vessel disease, *2VD* Two-vessel disease, *3VD* three-vessel disease, *CAD* coronary artery disease, *HDL-C* high density lipoprotein cholesterol, *LDL-C* low density lipoprotein cholesterol

^*^ *P*-values adjusted for diabetes mellitus, cigarette smoking, opium addiction, hypertension, and family history of CAD as well as body mass index, serum HDL-C, triglyceride, and LDL-C

^a^A total number of 505 patients after excluding 546 with normal coronary arteries (Gensini score = 0)

## Discussion

The importance of SCARB1 in reverse cholesterol transport and atherosclerosis has been previously confirmed in mouse models [11]. Moreover, recent studies have demonstrated rs5888 variant within SCARB1 to be linked with lipid levels and the size of lipoprotein particles, especially in younger women [23, 24]. However, the role of this variant in CAD development among humans is much less investigated [25]. Here we report, for the first time to our knowledge, that the SNP rs5888 in the SCARB1 gene is possibly an independent risk factor for early onset CAD in a cohort of young Iranian patients undergoing elective coronary angiography. In this study we focused on young patients with CAD because we wanted to investigate a cohort of patients who less affected by the age-related confounding factors; this strategy is more likely to detect genetic effects. The logistic regression model demonstrated that T allele in comparison with C allele had 1.3-fold increased risk for PCAD in whole and in women subgroup. Also, in men, the T allele showed a 20 % higher risk for CAD compared to C allele but this did not reach a statistical significance.

Among our study population, rs5888 within SCARB1 gene is a common SNP with minor allele frequency around 40 %. The variant allele of SNP rs5888 (T allele) frequency was 0.403 in total; 0.423 in case group and 0.386 in reference group. In a cross-sectional health survey on Lithuanian population, T allele frequency in the reference and MI groups was 0.362 and 0.400, respectively [18], whereas in Korean subjects the T allelic frequency was 0.330 in control and 0.170 in CAD [16]. In white population, the prevalence of this variant varies between 0.400 and 0.600 [13, 23, 26–28]. Among the participant of the Framingham Offspring Study, wherein almost all were Caucasian, the frequency of T allele was 0.486 [28], while in Chinese population T allele frequency varies from 0.217 to 0.290 [22, 29]. These studies suggest that the frequency of rs5888 mutation is different in various surveys with various sample sizes, in various geographical regions, and in various ethnic populations. Our finding that T allele may be a significant, independent risk factor for CAD is consistent with the results of a recent candidate gene association study on Chinese [14], but this is in contrast to the other similar studies [15–18]. In a Tunisian population, Rejab et al. [17] reported that subjects carrying T allele were associated with an almost 41 % reduced risk of angiographically documented significant coronary stenosis. Several other studies on Korean, Spanish, and Lithuanian patients also suggested that the T allele was associated with lower CAD risk [15, 16, 18].

Such disparity in study results may be ascribed to methodological heterogeneity (differences in study designs, sample sizes, definition of the phenotype, and, age and gender), different genetic background, and differences in the nature of various populations. For example, the precise definition of phenotype in studies on CAD association is of essential importance because individuals with significant CAD who are clinically silent may be classified as controls leading to a higher likelihood of null results. To avoid this potential bias in the current study, we defined phenotype based on objective angiographic documentation of coronary artery status. Notably, the examined polymorphism (rs5888) is not among the list of 46 loci that previously described to be robustly associated with CAD in large-scale genome-wide association studies (GWAS) [30, 31]. In the largest GWAS meta-analysis of CAD undertaken to date, the Coronary ARtery DIsease Genome wide Replication and Meta-analysis (CARDIoGRAM) plus The Coronary Artery Disease (C4D) consortium (CARDIoGRAMplusC4D), involving European and South Asian descent, 15 new susceptibility loci were identified [31]. Four of the 30 CAD risk loci previously reported in individuals of European ancestry (CDKN2B-AS1, COL4A2, CXCL12 and APOE) were detected as independent additional SNPs not in LD (r^2^ < 0.5) with the lead SNP, which also reached genome-wide significance. The additional SNP in the APOE locus was rs445925 (*P* = 9.42 × 10^−11^; r^2^ = 0.015 with rs207560 in 1000 Genomes Project data) [31]. There was no trend for higher odds ratios (ORs) in any of the subgroup analyses; however, one new locus reached genome-wide significance in males and in young CAD cases (rs16986953; *P* = 1.89 × 10^−8^ and 1.67 × 10^−8^, respectively), which was located in a gene desert (with nearest transcript AK097927), 1.3 Mb away from the APOB gene [31].

Even though several studies having suggested association between the genotypes of rs5888 gene polymorphism and various clinical parameters, the causative role of this variant is still unknown. Indeed, the exon 8 variant (rs5888) is a common synonymous or silent SNP that does not encode for a change in amino acid sequence of the protein product. Although this silent polymorphism was conventionally believed to be a nonfunctional marker in linkage disequilibrium with an as-yet-unidentified causal variant [13, 15, 32], no linkage has been found between this SNP and a known functional promoter variant [15]. Also, the promoter variant appears to be extremely rare (0.020) in white populations [15]. On the other hand, recent accumulating evidence indicates that silent SNPs could directly influence expression and/or function of their gene products [33–35]; using in vitro models, Constantineau et al. [12] demonstrated that synonymous rs5888 variant affected the RNA secondary structure and conformation of its gene product, SR-BI, and was significantly associated with lower SR-BI expression and its functionality. Hence, SCARB1 rs5888 T allele may possibly alter SR-BI expression, and in turn, influence risk for development of CAD. Moreover, another possible mechanism for this association may be related to some splicing regulatory role [36].

We found no associations between SCARB1 rs5888 (C > T) genotypes and lipid profile in our studied patients. In two other studies, the investigators also did not find such associations, when combined with other polymorphisms in the SCARB1 gene, the combined genotypes were related to triglyceride and HDL-C in the women [13, 37]. However, the association between SCARB1 gene and lipid profile is complex and still debated [18]. Another notable issue is that subgroup analysis of men and women demonstrated significant different frequencies of rs5888 variant of SCARB1 gene exclusively in women. Significant sex-dependent associations between SCARB1 genetic variations with lipid profile, BMI, and risk of cardiovascular disease have been previously shown [13, 17, 18, 27, 32]. As noted by Ritsch et al. [32], this phenomenon may be justified by a likely interaction between SCARB1 genotype and estrogen-dependent regulation of SR-BI expression and lipid parameters or by a possible effect of sex on factors affecting the function of SR-BI; factors such as cholesteryl gradient within the cells, HDL-C plasma concentration and composition of its particles, and triglyceride levels that influence HDL-C metabolism via cholesterol ester transfer protein.

There are several limitations in the present study that should be noted. First, because of case-control design, a selection bias may have been introduced. Second, the control group is not representative of the general healthy population, because it is enriched for people with a high clinical suspicion of having significant CAD, and although coronary stenosis is ruled out, cardiovascular risk factors can be assumed to be higher in this group as compared to the general population. Third, although we tested a relatively large number of patients with PCAD, our study was under-powered to detect the 1.2-fold effect size in male subjects with risk allele (T). Fourth, our results from a single-center may not be generalized to other ethnic groups; however, our tertiary referral center receives patients from all over the country with various ethnic backgrounds. Finally, the lack of ethnicity data in the present study is a major limitation that might explain some of the discrepancy with other studies particularly because the population was from various ethnic backgrounds; therefore, caution needs to be exercised in drawing conclusions about the impact of the examined SNP on PCAD.

## Conclusion

Using angiographic data and thus refining the phenotype, we found that the SNP (rs5888) within SCARB1 is independently associated with PCAD in whole study population and in women, but no association was observed in men. However, the statistical power was not sufficient to detect an association in male subgroup. Further studies, particularly in those with PCAD, with larger sample size in sex subgroups are required to confirm such association in well-defined ethnic groups. Moreover, because many genes are involved in the development of CAD due to dyslipidemia, further investigations are needed to test the relationship between polymorphisms associated with lipid metabolism and the risk of CAD. In addition, the exact mechanism and biological basis of possible role of rs5888 variant in pathogenesis of CAD needs to be explained.
